# Nasal colonization of methicillin resistant *Staphylococcus aureus* in Ethiopia: a systematic review and meta-analysis

**DOI:** 10.1186/s12941-019-0324-y

**Published:** 2019-09-05

**Authors:** Alemayehu Reta, Abeba Mengist, Asnakew Tesfahun

**Affiliations:** Department of Medical Laboratory Science, College of Health Sciences, Debre Markos University, Debre Markos, Ethiopia

**Keywords:** Methicillin-resistant *Staphylococcus aureus*, Nasal colonization, Meta-analysis, Ethiopia

## Abstract

**Background:**

Methicillin-resistant *Staphylococcus aureus* (MRSA) is one of a medically important Gram-positive bacteria, which can be harboured majorly in the nasal cavity. Risk of consequent infection in a person colonized with *S. aureus* as well as MRSA upsurges with time and remains insistently increased. Hence, the objective of this meta-analysis was to determine the prevalence of *S. aureus* and MRSA nasal colonization in Ethiopia at large.

**Methods:**

PubMed, Google Scholar, Embase, Hinari, Sci Hub, Scopus, and the Directory of Open Access Journals were searched and a total of 10 studies have been selected for meta-analysis. Preferred Reporting Items for Systematic Reviews and Meta-Analyses (PRISMA) guidelines were used for the literature search strategy, selection of publications, data extraction, and the reporting of results for the review. All statistical analyses were performed using STATA version 11 software via random effects model. The pooled prevalence was presented in forest plots and figure with 95% CI.

**Results:**

A total of ten studies with 2495 nasal swab samples were included in this meta-analysis, and the overall pooled estimated prevalence of *S. aureus* and MRSA nasal colonization in Ethiopia were 30.90% [95% CI 21.81–39.99%], 10.94% [95% CI 8.13–13.75%] respectively. Subgroup analysis was also noted in different regions of Ethiopia, henceforth Oromia region ranked first 21.28% [95% CI 8.22–34.35%], followed by Amhara region 6.78% [95% CI 3.02–10.54%], whereas relatively low magnitude of MRSA colonization was demonstrated from Tigray region 4.82% [95% CI 2.18–7.45%].

**Conclusion:**

The analysis showed that the overall prevalence of *S. aureus* and MRSA nasal colonization in Ethiopia were comparable with the global prevalence. But a huge variation between the regions, so the Ministry of Health of Ethiopia should design appropriate decolonization program that can address the specific regional groups as well as the national population.

## Background

Methicillin-resistant *Staphylococcus aureus* (MRSA) is one of a medically important Gram-positive bacteria, which first emerged since 1961 [1]. It is introduced to the African continent in 1978 and appeared in Ethiopia in 1987 [2, 3]. Among the body sites that harbour MRSA such as throat, perineum, skin, hairline, groin, and the axilla, the anterior nares, the most important site for MRSA colonization is the nasal cavity [4]. Nasal colonization with *S. aureus* is a vibrant process; a number of factors being responsible for the gain and loss of carriage. Risk of consequent infection in a person colonized with *S. aureus* as well as MRSA upsurges with time and remains insistently increased [5].

MRSA is defined as any strain of *S*. *aureus* that has established resistance to beta-lactam antibiotics such as Methicillin, Oxacillin, Cefoxitin and Nafcillin [6, 7]. These strains are responsible for a greater number of nosocomial infections which are tough to combat in humans [8]. MRSA has been conveyed with alarming frequencies worldwide, and these strains majorly unveil multi-drug resistance, that is resistant to three or more classes of antibiotics [9]. Multidrug resistance of nasal *S*. *aureus* associated with methicillin-resistant strains is of great public health concern especially in developing countries [10].

Several studies in Ethiopia have reported the rate of nasal colonization of *S. aureus* and MRSA varying from 12% to 60% and 0% to 29% respectively. Increased carriage rate was recorded among patients in both *S. aureus* and MRSA [11–20].

Elimination of nasal carriage has been reported to cause a significant reduction in the incidence of MRSA infections in the community [21]. Understanding the overall epidemiology of nasal colonization of *S. aureus* and MRSA at the country level is so significant to strengthen effective prevention and control strategies. So, this meta-analysis was aimed to summarize available data and to determine the pooled prevalence of *S. aureus* and MRSA nasal colonization in Ethiopia by conducting a meta-analysis.

## Methods

### Study design and literature search strategy

This study did a meta-analysis on nasal colonization of *S. aureus* and MRSA in Ethiopia using the best available studies. Preferred Reporting Items for Systematic Reviews and Meta-Analyses (PRISMA) guidelines were used for the literature search strategy, selection of publications, data extraction, and the reporting of results for the review [22]. Published works of literatures on nasal colonization of MRSA were reviewed using the following major databases; PubMed, Google Scholar, Embase, Hinari, Sci Hub, Scopus, and the Directory of Open Access Journals (DOAJ) and additional articles identified through other electronic sources (Google, Cochrane library and reference lists). Literature searches were conducted from July to September 2018. During a comprehensive literature search, the following search terms were used: “nasal colonization, nasal carriage, Methicillin-Resistant *Staphylococcus aureus* (MRSA), *Staphylococcus aureus*, *S. aureus*, Ethiopia’’. Various combinations of key terms were used through Boolean search technique. Additionally, the references cited by each eligible study were scrutinized to identify additional articles. We did not limit the search by a year of publication.

### Study selection and eligibility criteria

Inclusion criteria: In this meta-analysis, we included all studies that were conducted on nasal colonization of MRSA in Ethiopia. All available studies and data were incorporated based on the following predefined eligibility criteria: Should be published and written in English, had to describe the standard microbial isolation and identification, studies should use human originated sample and should be a prospective cross-sectional study.

Exclusion criteria: Studies that used samples other than human origin and nasal swab; Studies that didn’t describe the standard microbial isolation and identification techniques and studies with no full information to calculate the prevalence MRSA were excluded.

### Outcome of interest

The major outcome of interest was the prevalence of MRSA and *S. aureus* nasal swab isolates. The prevalence was calculated by dividing both the numbers of MRSA and *S. aureus* isolates to the total number of nasal swab samples.

### Quality assessment and critical appraisal

In this meta-analysis, the qualities of each article were assessed by using a critical appraisal tool for use in systematic reviews for prevalence study [23]. The employed methods for MRSA isolation and eligibility of the identified articles were also assessed by all authors and disagreements among reviewers were fixed accordingly with a discussion. In addition, a modified version of the Newcastle–Ottawa Scale for the cross-sectional study was used to evaluate the quality of studies [24].

### Data extraction

Data from appropriate studies were pull out independently by authors and potted into a spreadsheet. Inconsistencies were resolved by unanimity. For each of the encompassed studies, the following information was extracted; the name of the author, publication year, study area, study period, study design, study population, number of samples, number of *S. aureus* isolates, number of MRSA, the prevalence of *S. aureus* and prevalence of MRSA.

### Data analysis and synthesis

The extracted data were entered into the computer through command window of STATA software version 11 and the data manipulation and all statistical analysis were performed using STATA version 11. A random effect model was used to estimate the overall pooled prevalence of *S. aureus* and MRSA and this model was recommended to handle heterogeneity between studies for meta-analysis [25–27]. The I^2^ statistical test was used to check heterogeneity, and it quantified the percentage of total variation in the study [28]. I^2^ test ranges from 0% (observed heterogeneity) to 100% (significant heterogeneity). A p value < 0.05 was used to declare heterogeneity [29, 30]. In the current meta-analysis, I^2^ values were found to be high (> 75%). This scenario leads to use a random effects model with 95% CIs to avoid the significant heterogeneity. Furthermore, the presence of heterogeneity was also assessed by subgroup analysis and meta-regression. Visual assessment of publication bias was shown using funnel plot. Asymmetry of the funnel plot is an indicator of publication bias [22]. To check potential publication bias (small study bias), Egger’s and Begg’s tests were performed [31]. Also, the sensitivity analysis was done to assess whether the pooled prevalence estimates were prejudiced by individual studies.

## Result

### Selection and identification of studies

**Fig. 1:**
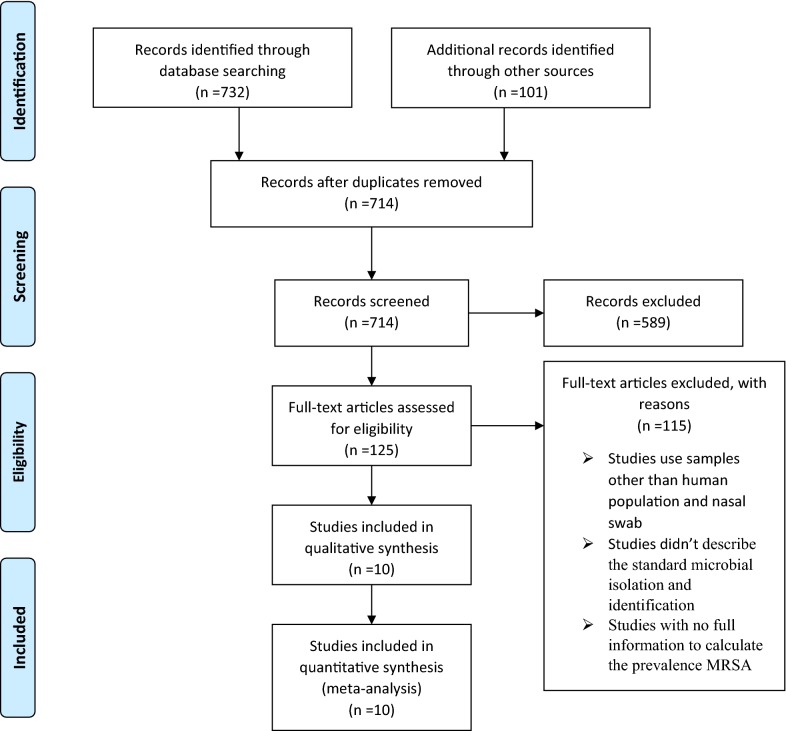
Flowchart shows study selection for meta-analysis of nasal colonization of MRSA in Ethiopia, 2018

The process of identifying the relevant articles for inclusion in this meta-analysis is represented graphically in Fig. 1. A total of 732 studies were identified through an electronic database search. We added 101 additional articles identified through other electronic sources (Google, Cochrane library and reference lists). Of these studies, 589 were excluded after reviewing their title and abstracts, 119 were found to be duplicates, and 115 were disregarded because the abstracts or full-text information did not directly relate to the topic of interest i.e. nasal colonization of MRSA in Ethiopia. Finally, 10 unique articles fulfilled our eligibility criteria and were enrolled for meta-analysis (Fig. 1).

### Characteristics of included studies

A total of ten studies with 2495 nasal swab samples were included in this meta-analysis was summarized in Table 1. The studies were conducted from 2003 to 2018 in different regions of the country. Among 10 studies, four of them [11, 16, 18, 19] were conducted in Amhara region, three studies [12, 14, 20] were in the Oromia region, and the other three studies [13, 15, 17] were in Tigray regional state of the country, no data was obtained from other regions (Benishangul-Gumuz, Gambella, Somali, SNNP (Southern Nations Nationalities and People), Afar, Dire Dawa and Addis Ababa). All studies were a cross-sectional study conducted on nasal colonization of MRSA in Ethiopia. The study with a minimum and a maximum nasal swab sample was conducted in Oromia and Amhara region, respectively [14, 18]. In addition, out of all studies enrolled in this meta-analysis eight studies [11, 13–16, 18–20] were conducted among patients while the remaining two studies [12, 17] were conducted from apparently healthy individuals (Table 1).

**Table 1:** Characteristics of studies included in meta-analysis of nasal colonization of MRSA in Ethiopia, 2018

S/No	Author	Publication year	Region	Study area	Study period	Study design	Study population	No of sample	No of *S. aureus* isolates	Prevalence of S*. aureus* (%)	No of MRSA isolates	Prevalence of MRSA (%)	References
1	Shibabaw et al.	2013	Amhara	Dessie	November 2010 to March 2011	Cross sectional	Healthcare workers	118	34	28.8	15	12.7	[11]
2	Dagnew et al.	2012	Amhara	Gonder	January 1, 2011 to June 30, 2011	Cross sectional	Food handlers	200	41	20.5	4	2	[16]
3	Reta et al.	2017	Amhara	Debre Markos	April to June, 2015	Cross sectional	Pre-school children	400	52	13	0	0	[18]
4	Reta et al.	2014	Amhara	Bahir Dar	March 1 to June 30, 2013	Cross sectional	School children	300	123	41	17	5.67	[19]
5	Balta et al.	2003	Oromia	Jimma	January 22 to February 18, 2002	Cross sectional	Inpatients	152	85	55.9	44	28.9	[12]
6	Kejela et al.	2013	Oromia	Jimma	December 2010 to March 2011	Cross sectional	School children and prisoners	354	169	47.7	39	11	[20]
7	Elemo et al.	2017	Oromia	Asella	November 2012–May 2013	Cross sectional	Dairy workers	96	38	39.6	23	23.96	[14]
8	Legese et al.	2018	Tigray	Adigrat Wukro	September to December 2016	Cross sectional	Healthcare workers	242	29	12	14	5.8	[13]
9	Gebremedhn et al.	2016	Tigray	Mekelle	September 2014 to February 2015	Cross sectional	HIV patients	249	81	32.5	6	2.4	[17]
10	Kahsay et al.	2018	Tigray	Mekelle	January to May 2016	Cross sectional	Janitors	384	69	18	24	6.25	[15]

### Nasal colonization of *S. aureus*

**Fig. 2:**
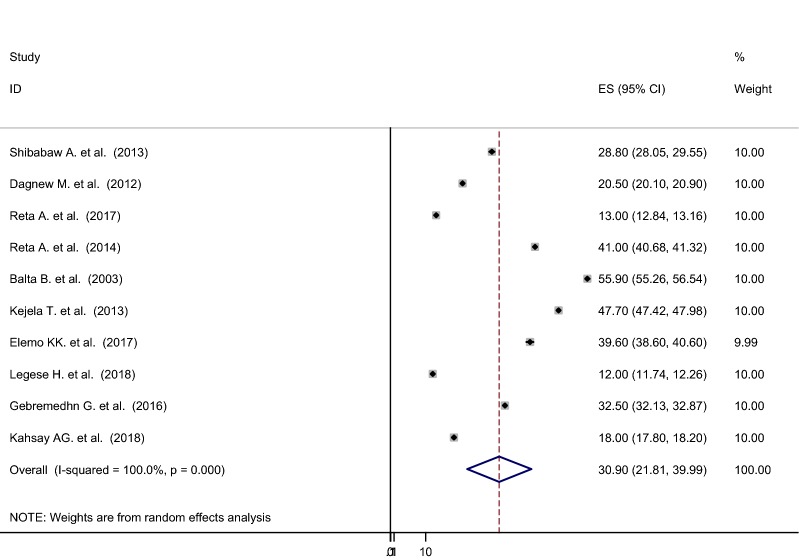
Forest plot showing the pooled prevalence of nasal colonization of *S. aureus* in 10 studies in Ethiopia, 2018

**Fig. 3:**
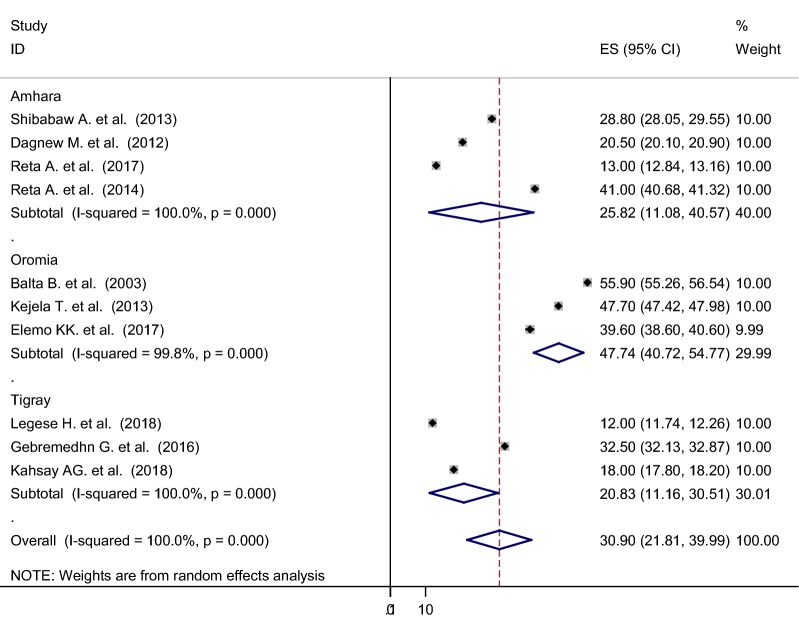
Subgroup analysis by regions on the prevalence of nasal colonization of *S. aureus* in 10 studies in Ethiopia, 2018

The pooled prevalence using the fixed effect model revealed significant heterogeneity between the studies. Hereafter, we performed the analysis using the random effects model. Using random effects model, the estimated pooled prevalence of nasal colonization of *S. aureus* stated by the ten studies was 30.90% [95% CI 21.81–39.99%] with significant heterogeneity between studies (I^2^ = 100%, p < 0.001). The pooled prevalence of nasal colonization of *S. aureus* offered using forest plot (Fig. 2). To assess the potential heterogeneity between studies, subgroup analysis by study area was conducted. Of the ten studies, the highest expected nasal colonization of *S. aureus* prevalence found in studies showed in Oromia region 47.74% [95% CI 40.72–54.77%], I^2^ = 99.8%, p < 0.001, followed by studies showed in Amhara region, was 25.82% [95% CI 11.08–40.57%], I^2^ = 100%, p < 0.001 (Fig. 3).

### Nasal colonization of MRSA

**Fig. 4:**
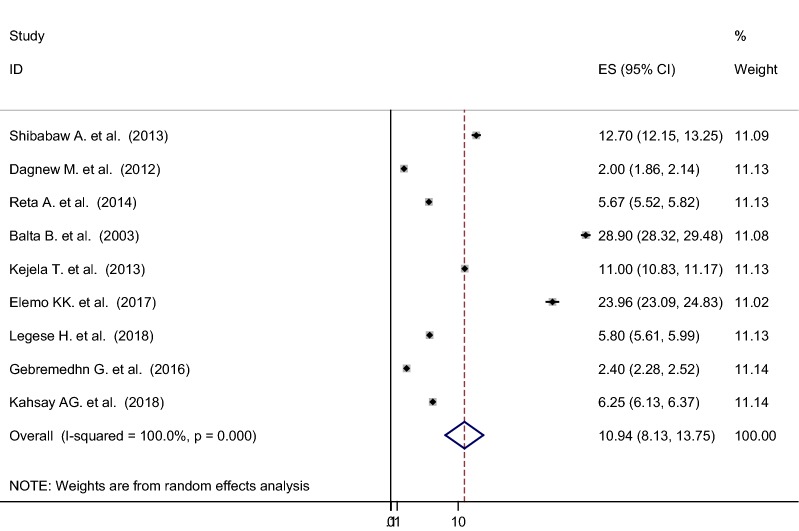
Forest plot showing the pooled prevalence of nasal colonization of MRSA in 9 studies in Ethiopia, 2018

**Fig. 5:**
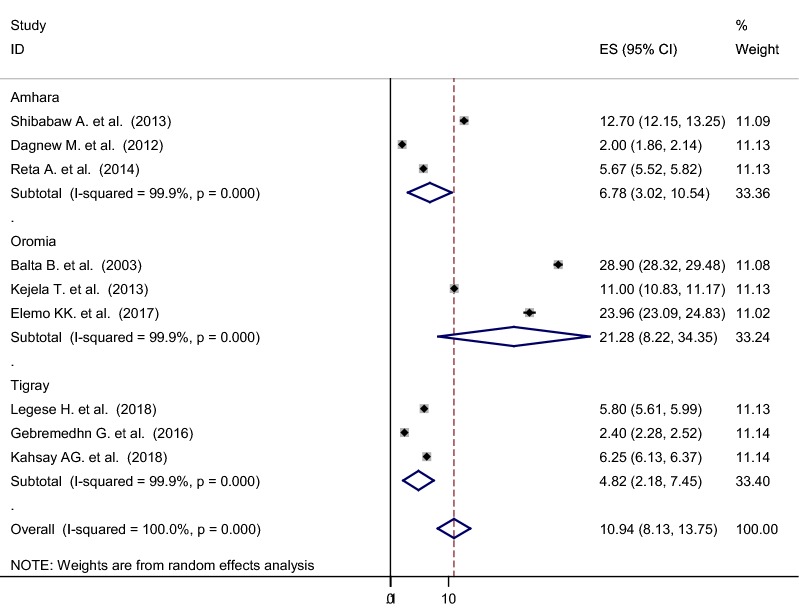
Subgroup analysis by regions on the prevalence of nasal colonization of MRSA in 9 studies in Ethiopia, 2018

**Fig. 6:**
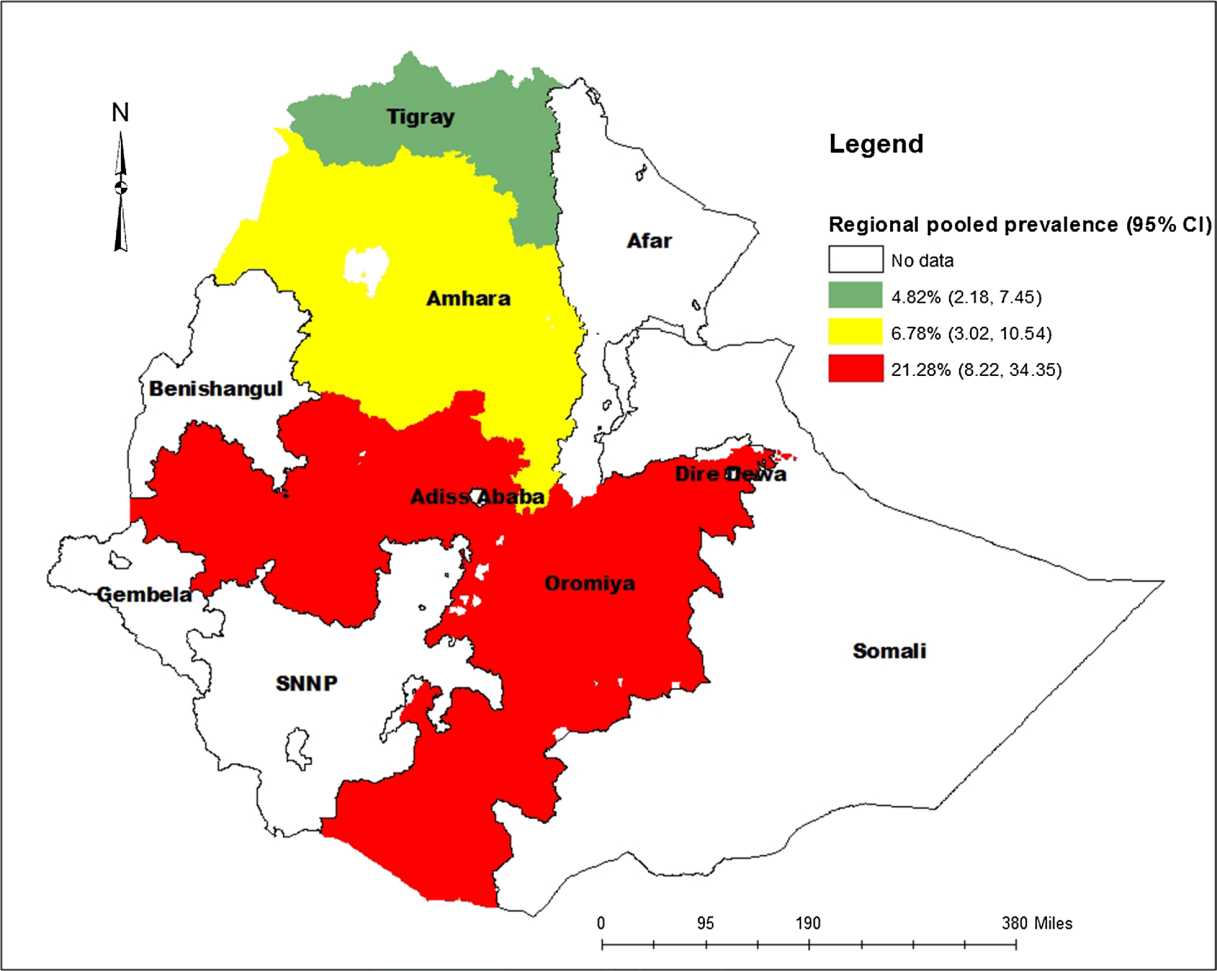
Prevalence of nasal colonization of MRSA in different regions of Ethiopia, 2018

As shown from Fig. 4, the overall pooled prevalence of nasal colonization of MRSA was 10.94% [95% CI 8.13–13.75%], I^2^ = 100%, p < 0.001. Subgroup analysis was also noted in different regions of Ethiopia, henceforth Oromia region ranked first 21.28% [95% CI 8.22–34.35%], I^2^ = 99.9%, p < 0.001, followed by Amhara region 6.78% [95% CI 3.02–10.54%], I^2^ = 100%, p < 0.001, whereas relatively low magnitude of MRSA colonization were demonstrated from Tigray region 4.82% [95% CI 2.18–7.45%], I^2^ = 100%, p < 0.001 (Figs. 5 and 6).

### Investigation of heterogeneity

In this meta-analysis, the value of I^2^ indicates a significant high heterogeneity, so we adjust this by using random effect model and the source of heterogeneity was investigated using meta-regression model using publication year and the number of a sample as covariates. The result of meta-regression analysis revealed that both covariates were not statistically significant for the presence of heterogeneity (Table 2).

**Table 2:** Meta-regression analysis of factors with heterogeneity of the prevalence of nasal colonization of *S. aureus* in Ethiopia, 2018

Heterogeneity source	Coefficients	Std. err.	t	P value	95% conf. interval	
Publication year	− 2.358079	2.81955	− 0.84	0.431	− 9.025255	4.309097
No of sample	− 0.0114633	0.077581	− 0.15	0.887	− 0.1949132	0.1719866

### Publication bias

**Fig. 7:**
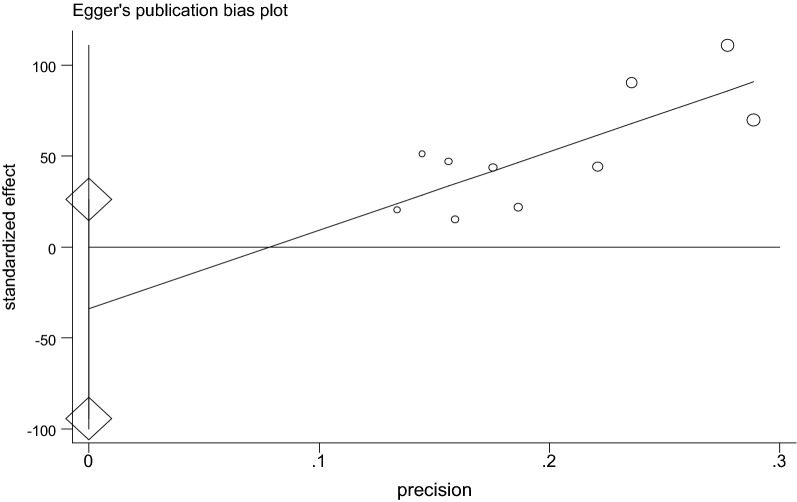
The result of Egger’s publication bias test of 10 studies on nasal colonization of *S. aureus*, 2018

**Fig. 8:**
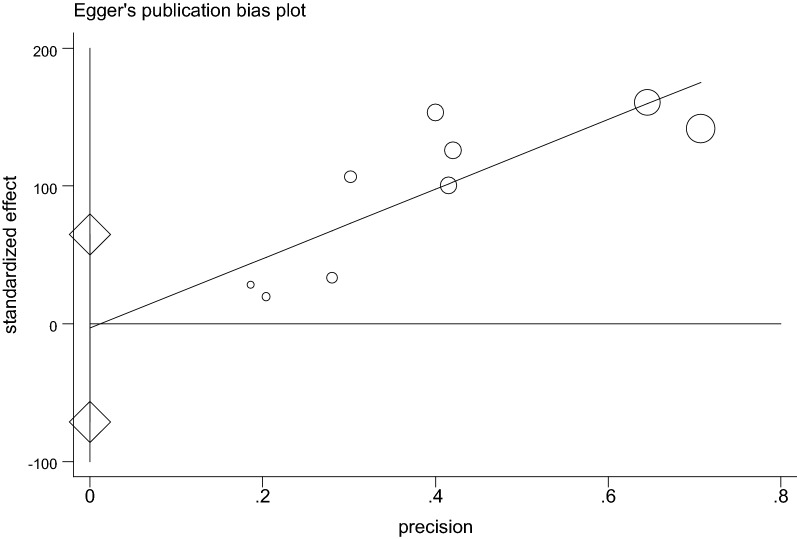
The result of Egger’s publication bias test of 9 studies on nasal colonization of MRSA, 2018

The presence of publication bias was assessed using Begg’s and Egger’s tests, showing no statistical significance for estimating the prevalence of nasal colonization of *S. aureus* and MRSA (Table 3, Figs. 7 and 8).

**Table 3:** P-value for Begg’s and Egger’s test for nasal colonization of *S. aureus* and MRSA, 2018

Tests	P value	
	Nasal colonization of *S. aureus*	Nasal colonization of MRSA
Begg’s	0.721	0.251
Egger’s	0.353	0.167

### Sensitivity analysis

**Fig. 9:**
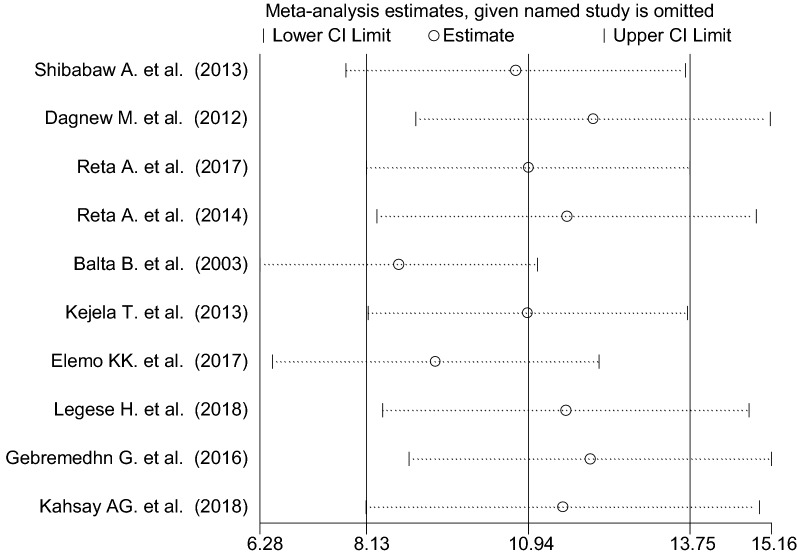
Result of sensitivity analysis of the studies on nasal colonization of MRSA, 2018

**Fig. 10:**
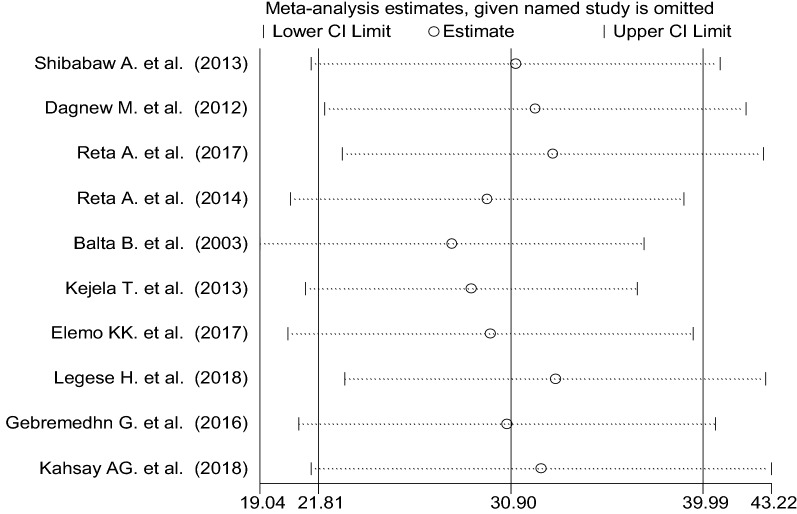
Result of sensitivity analysis of the studies on nasal colonization of *S. aureus*, 2018

The result of sensitivity analysis using random effects model suggested that no single study improperly influenced the overall prevalence estimate of nasal colonization of *S. aureus* and MRSA (Figs. 9 and 10).

## Discussion

We conducted this meta-analysis to estimate the overall pooled prevalence of nasal colonization of *S. aureus* and MRSA in Ethiopia. Ten unique articles which fulfilled our eligibility criteria were included and enrolled for meta-analysis. After analysis, the overall estimated pooled prevalence of nasal colonization of *S. aureus* and MRSA in Ethiopia were 30.90% [95% CI 21.81–39.99%]), 10.94% [95% CI 8.13–13.75%]) respectively. The overall prevalence of nasal colonization of *S. aureus* indicated in this meta-analysis is in line with a meta-analysis conducted in Iran (22.7% [95% CI 19.3–26.6]). The pooled estimate prevalence of nasal colonization of MRSA in this meta-analysis is lower than a meta-analysis conducted in Iran (32.8% (95% CI 26.0–40.4) [32] and Egypt (32%) [33], and relatively higher than a systematic review done in Europe and the United States of America (1.8% [95% CI 1.34–2.50%]) [34], Moreover, it is in agreement with a study done in Nigeria (11%) [35]. The possible explanations for the above variations could be due to methodological differences (i.e., Microbiological isolation and detection techniques), variation in sociodemographic characteristics, economic, and health service utilization.

In this study, we also performed subgroup analysis based on the regions of the country where the studies were conducted. The findings of the subgroup analysis indicated that extreme variability was observed in both the prevalence of nasal colonization of *S. aureus* and MRSA across the regions of the country. The highest (47.74%, 21.28%) prevalence of nasal colonization of *S. aureus* and MRSA respectively was reported from the Oromia region, whereas the lowest (20.83%, 4.82%) prevalence of nasal colonization of *S. aureus* and MRSA was reported from Tigray region. The possible explanation for this variation might be due to the cultural variation, the difference in health infrastructure and health policy implementation across the regions of the country.

## Limitations of the study

Due to the absence of similar and related factors, we were unable to perform factor analysis and examine the pooled odds ratios, even if there are a number of factors in each study such as age, sex, socioeconomic status, use of antibiotics, hospitalization, recurrent acute otitis media, hygienic practice of the hand and the like. However, this review has delivered valuable information concerning the pooled estimate prevalence of nasal colonization of *S. aureus* and MRSA.

## Conclusions and recommendations

This meta-analysis demonstrates that the pooled estimated prevalence of nasal colonization of *S. aureus* and MRSA in Ethiopia was relatively in a comparable prevalence with the total nasal colonization of *S. aureus* and MRSA as a whole in the human population. But, when we observed the pooled estimate prevalence of each (*S. aureus* and MRSA) in different regions of Ethiopia, it has extreme variation. Thus, to contest the burden of nasal colonization of *S. aureus* and MRSA in particular, the following concerns should be well thought-out at the national level, such as adopting safety protocols and implementing proper decolonization policies in a regional base.

Finally, in Ethiopia, conducting such type of study will give additional input for program planners and policy makers working in the area of infectious disease and also it will help clinicians to give empirical treatment.

## Data Availability

Data sharing is not applicable to this article as no datasets were generated or analyzed during the current study.

## Abbreviations

CI: confidence interval
DOAJ: Directory of Open Access Journals
MRSA: methicillin-resistant *Staphylococcus aureus*
PRISMA: Preferred Reporting Items for Systematic Reviews and Meta-Analyses
SNNP: Southern Nations Nationalities and People
STATA: statistics and data
